# The Clinical Significance of Salusins in Systemic Sclerosis—A Cross-Sectional Study

**DOI:** 10.3390/diagnostics13050848

**Published:** 2023-02-23

**Authors:** Joanna Nowaczyk, Leszek Blicharz, Michał Zawistowski, Mariusz Sikora, Michał Zaremba, Joanna Czuwara, Lidia Rudnicka

**Affiliations:** 1 Department of Dermatology, Medical University of Warsaw, 02-091 Warsaw, Poland; 2 National Institute of Geriatrics, Rheumatology and Rehabilitation, 02-637 Warsaw, Poland

**Keywords:** biomarkers, inflammation, fibrosis, systemic sclerosis, vascular remodeling, vasodilatation

## Abstract

**Background:** Systemic sclerosis (SSc) is a connective tissue disease manifesting with progressive fibrosis of the skin and internal organs. Its pathogenesis is strictly associated with vascular disfunction and damage. Salusin-α and salusin-β, endogenous peptides regulating secretion of pro-inflammatory cytokines and vascular smooth muscle proliferation, may potentially play a role in SSc pathogenesis. **Objectives:** The aim of this study was to assess the concentration of salusins in sera of patients with SSc and healthy controls and to evaluate correlations between the salusins levels and selected clinical parameters within the study group. **Materials and methods:** 48 patients with SSc (44 women; mean age, 56.4, standard deviation, 11.4) and 25 adult healthy volunteers (25 women; mean age, 55.2, standard deviation, 11.2) were enrolled. All patients with SSc were treated with vasodilators and twenty-seven of them (56%) also received immunosuppressive therapy. **Results:** Circulating salusin-α was significantly elevated in patients with SSc in comparison to healthy controls (U = 350.5, *p* = 0.004). Patients with SSc receiving immunosuppression had higher serum salusin-α concentrations compared with those without immunosuppressive therapy (U = 176.0, *p* = 0.026). No correlation was observed between salusins concentrations and skin or internal organ involvement parameters. **Conclusions:** Salusin-α, a bioactive peptide mitigating the endothelial disfunction, was elevated in patients with systemic sclerosis receiving vasodilators and immunosuppressants. Increased salusin-α concertation may be associated with the initiation of atheroprotective processes in patients with SSc managed pharmacologically, which requires verification in future studies.

## 1. Introduction

Systemic sclerosis is an autoimmune connective tissue disease characterized by progressive fibrosis of the skin and internal organs. Microvascular changes with endothelial disfunction, epigenetics, and complex autoimmune responses are the major factors contributing to the development of the disease [1]. In systemic sclerosis, vascular damage plays a pivotal role in the pathogenesis of tissue fibrosis; therefore, identifying markers of endothelial disfunction is crucial to help optimize patients assessments and to develop novel treatment methods aimed at mitigating progressive fibrosis [2,3,4,5]. One of the therapeutic aims is to stabilize the progression of microangiopathy, i.e., the loss of capillaries over time, which correlates with the course of disease [6]. Despite that circulating antibodies, such as Scl-70 (anti-topoisomerase I), are known to be associated with disease prognosis [7], limited evidence is available in regard to reliable therapeutic biomarkers in systemic sclerosis [8]. Therefore, researchers are constantly investigating new molecules of potential significance in the pathogenesis of the disease.

### 1.1. Bioactive Peptide Biomarkers-Salusin-α and Salusin-β

Among the candidates to be considered as systemic sclerosis biomarkers are salusins. These are two bioactive peptides, named salusin-α and salusin-β, which are derived from the TOR2A precursor, preprosalusin [9,10]. Salusins are produced in kidneys, central nervous system, and cardiovascular system [9,10]. In vitro studies have shown that salusin-α has anti-inflammatory properties manifested by the ability to decrease the expression of pro-inflammatory interleukins, i.e., IL-6, IL-8, and IL-18 [11]. Salusin-β promotes vascular remodeling as a result of various processes, involving vascular smooth muscle cell proliferation (the cAMP–PKA–EGFR-CREB/ERK pathway), fibrosis (the TGF-β1-Smad pathway), the activation of NF-κB pathway with reactive oxygen species (ROS) production, and increased mRNA expression of collagen I, collagen III, fibronectin, TGF-β1, and connective tissue growth factor [12,13,14,15,16]. 

### 1.2. Salusins and the Vascular Tone Regulation

Due to its involvement in vascular homeostasis pathways, salusins may play a role in fibroproliferative vasculopathy in systemic sclerosis. One of the important factors in vascular tone regulation is disbalance between nitric oxide and endotelin-1 (ET-1), both secreted by the endothelium, which may be relevant for the progression of endothelial disfunction in patients with systemic sclerosis [5,17]. A vasoconstrictor peptide, endothelin-1, modulates the vascular tone to maintain the proper vascular homeostasis by the mediation of smooth muscle vasoconstriction (through the ET_A_ receptor) and stimulation of nitric oxide secretion (through the ET_B_ receptor) [5,18,19]. Salusin-β was found to attenuate vasodilation in the mice model of hypertension by activating reactive oxygen species and inhibiting nitric oxide release [20,21]. In rats with induced pulmonary arterial hypertension, salusin-β inhibition contributed to decreased macrophage infiltration and pro-inflammatory cytokine expression in the lungs [22]. 

### 1.3. Salusins and the Endothelial Disfunction

The dysregulation of endothelin-1/nitric oxide ratio may also contribute to cardiovascular disorders associated with endothelial disfunction, such as atherosclerosis and arterial hypertension [18]. In the process of tissue fibrosis, vascular myofibroblasts may undergo cellular differentiation referred to as endothelial-to-mesenchymal transition, which is a conversion of the cell phenotype from endothelium markers into mesenchymal cell markers [5]. In a murine model, salusin-α injections were associated with decreased TNFα and IL-6 levels, arterial plaque formation, and a decreased presence of foam cells, while salusin-β was associated with increased TNFα and IL-6 concentrations, atherogenesis, and foam cell formation [14,23]. The impact of salusin-β on arterial plaque formation is related to the NF-κB pathway; however, salusin-α is not involved in this pathway [11,14,24]. Salusin-α mitigates atherosclerosis through the inhibition of vascular smooth muscle cells proliferation and migration mediated by the Akt/mTOR pathway [25].

## 2. Materials and Methods

A retrospective single-center pilot cross-sectional study was conducted at the Department of Dermatology, Medical University of Warsaw, Poland. Its results were reported in line with the strengthening the reporting of observational studies in epidemiology (STROBE) statement [26]. The primary aim of this study was to compare the concentrations of salusin-α and salusin-β in sera of patients with systemic sclerosis and healthy controls. Furthermore, exploratory analyses were performed in the systemic sclerosis group, only to find potential associations between serum salusin-α and salusin-β levels and selected clinical parameters reflecting skin or internal organ involvement in the disease.

### 2.1. Ethics

The study protocol was approved by the institutional review board, the Ethics Committee at the Medical University of Warsaw (the study protocol approval number KB/149/2020). The study was completed in accordance with the ethical standards of the 1964 Helsinki declaration with future revisions and international conference on harmonization good clinical practice guidelines.

### 2.2. Study Group and Data Collection

Adult patients with systemic sclerosis (diffuse or localized) consecutively admitted between May 2019 and July 2021 to the Department of Dermatology, Medical University of Warsaw, Poland were considered eligible for inclusion in this study. Patients were undergoing their treatment in accordance with the current recommendations of the Polish Dermatological Society [27]. Only individuals who were electively admitted in order to receive intravenous vasodilative treatment (alprostadil, epoprostenol, or sulodexide) and who signed an informed consent form were enrolled. Exclusion criteria were active cancer, infection, pregnancy, chronic systemic treatment with nonsteroidal anti-inflammatory drugs or corticosteroids, and any modification of systemic therapy within the preceding month. 

In this study, a review of inpatient medical records was the main source of demographical and clinical data. All participants had their blood samples collected for further evaluation, including salusin-α and salusin-β concentration assessment. Other tests were performed as part of their routine clinical assessment. For patients in the experimental (systemic sclerosis) group, demographic information and medical details were collected, including current treatment and comorbidities. Findings in the recent imaging examinations were retrieved, such as high-resolution computed tomography (HRCT) of the thorax performed within the last year, diffusion lung capacity for carbon monoxide (DLCO) value assessed within the last year, and the esophageal X-ray with contrast from the previous three years. Complete blood count test results (including the counts of lymphocytes and neutrophils used to calculate the neutrophil-to-lymphocyte ratio; NLR), serum creatinine levels (used to calculate estimated glomerular filtration rate with the Chronic Kidney Disease Epidemiology Collaboration 2021 equation [28,29]), erythrocyte sedimentation rate (ESR) values, and C-reactive protein (CRP) concentrations were determined in the samples collected on the same day as the samples for salusins concentration assessment. Occurrence of fingertip erosions and the severity of skin thickening according to the modified Rodnan skin score (mRSS) [30] were assessed at the time of elective admission to the ward. 

The control group consisted of healthy volunteers, from whom only demographic details (age and BMI) were collected apart from the blood samples used for the determination of their salusin-α and salusin-β concentrations. Individuals from the control group were not diagnosed with metabolic diseases and did not have past or present history of glucocorticoids treatment or other immunosuppressive medications. 

### 2.3. Salusin-α and Salusin-β Concentrations Measurement

Salusin-α and salusin-β levels were analyzed at the Laboratory of Immunodermatology, Department of Dermatology, Medical University of Warsaw, Poland. Enzyme-linked immunosorbent assay kits (Bioassay Technology Laboratory, Shanghai, China) were used as per the manufacturer’s protocol.

### 2.4. Statistical Analysis

For the purpose of descriptive analysis, continuous variables were evaluated using histograms, the Shapiro–Wilk test and Q-Q plot analysis. Normally distributed variables were reported using means and standard deviations, while non-normally distributed ones were summarized with medians and ranges. Qualitative variables were described using the numbers of events and frequencies. To compare differences in continuous variables between the groups, Student’s t-test for normally distributed or Mann–Whitney U test for non-normally distributed parameters were used. The assumption of equal variances was verified with the Levene’s test. For the comparison of categorical variables with all expected values of at least 5, the Chi-squared test was utilized. Otherwise, the Fisher’s exact test was applied. Simple linear regression was used to assess the relationship between serum salusin-α and salusin-β concentrations in patients with systemic sclerosis, healthy volunteers and both groups combined. To find correlations or associations between preselected variables, Spearman’s ranked correlation and Gaussian graphical model analysis were used (as part of the exploratory analysis). All instances of missing data were assumed to be missing completely at random and deleted pairwise. No data were imputed. Two-sided *p* values < 0.05 were considered statistically significant. No correction for multiple comparisons was applied. All calculations were completed and plots created using the psych [31], the qgraph [32], the seolmatrix [33], and the ppcor [34] modules in the statistical software jamovi 2.3.21 (The jamovi project, 2022) [35,36].

## 3. Results

Forty-eight patients with systemic sclerosis (44 women; mean age, 56.4 years, standard deviation, 11.4) and twenty-five healthy volunteers (25 women; mean age, 55.2 years, standard deviation, 11.2; mean body mass index [BMI], 23.6, standard deviation, 4.2) were enrolled in this study. Detailed characteristics of the study group are presented in Table 1. There were no statistically significant differences in sex (*p* = 0.292), age (t[71] = 0.44, *p* = 0.664), and BMI (t[68] = 1.29, *p* = 0.201) between the experimental and control groups.

### 3.1. Salusin-α and Salusin-β Concentrations

**Figure 1:**
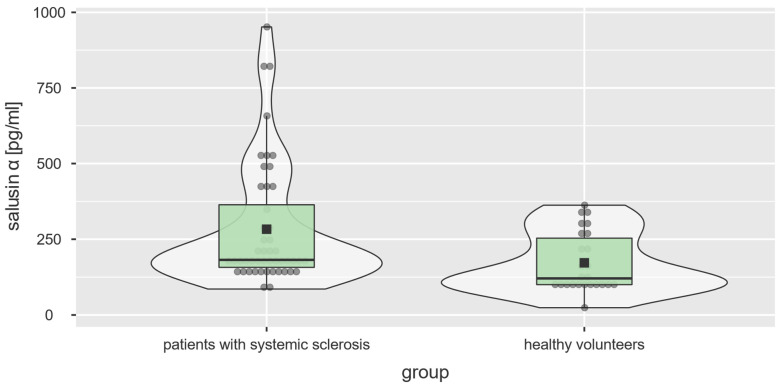
Salusin-α concentrations [pg/mL] in the experimental and control groups (U = 350.5; *p* = 0.004).

**Figure 2:**
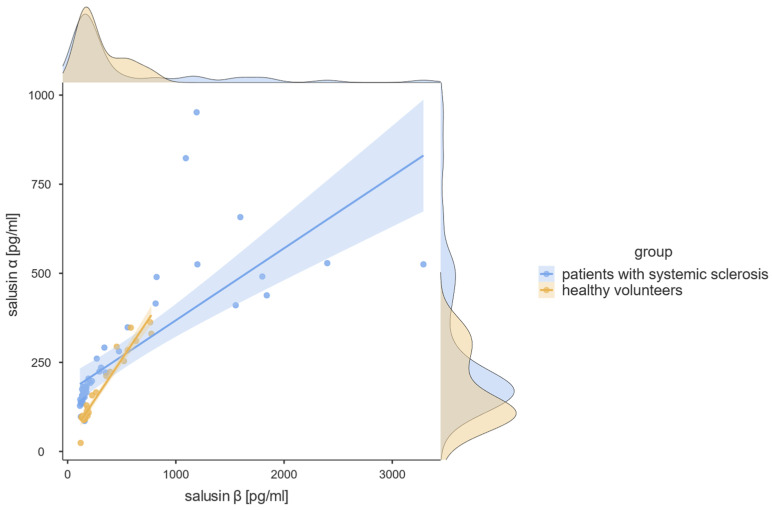
Simple linear correlation between salusin-α and β concentrations [pg/mL]. Statistically significant linear regression results were found. For all patients: F(1, 70) = 94.56; *p* < 0.001; R^2^ = 0.57, for the systemic sclerosis group: F(1, 45) = 54.81; *p* < 0.001; R^2^ = 0.55, for the healthy volunteers: F(1, 23) = 326.64; *p* < 0.001; R^2^ = 0.93.

Salusin-α was significantly elevated in patients with systemic sclerosis when compared with healthy volunteers (181.85 [85.66–951.80] vs. 120.70 [24.03–362.40] pg/mL; U = 350.5; *p* = 0.004; Table 2, Figure 1). No such association was detected for salusin-β (173.70 [113.70–3288.00] vs. 186.30 [120.30–773.50]; U = 550.0; *p* = 0.662). Salusin-α and β concentrations were strongly correlated with each other (F[1, 70] = 94.56, *p* < 0.001, R^2^ = 0.57, Figure 2).

### 3.2. Anti-Topoisomerase I (Scl-70) and Anti-Centromere (ACA) Antibodies

No statistically significant associations were detected between the presence of Scl-70 (anti-topoisomerase I) or ACA and salusin-α or β concentrations (for salusin-α and Scl-70, 174.5 [97.4–951.8] vs. 186.7 [85.7–525.1] pg/mL, U = 264.5, *p* = 0.815; for salusin-α and ACA, 180.0 [128.2–823.0] vs. 187.2 [85.7–951.8] pg/mL, U = 254.5, *p* = 0.812; for salusin-β and Scl-70, 160.0 [116.9–1840.0] vs. 178.4 [113.7–3288.0] pg/mL, U = 234, *p* = 0.516; for salusin-β and ACA, 170.3 [113.7–1092.0] vs. 174.5 [121.2–3288.0] pg/mL, U = 213.5, *p* = 0.343).

### 3.3. Skin and Internal Organ Involvement

**Figure 3:**
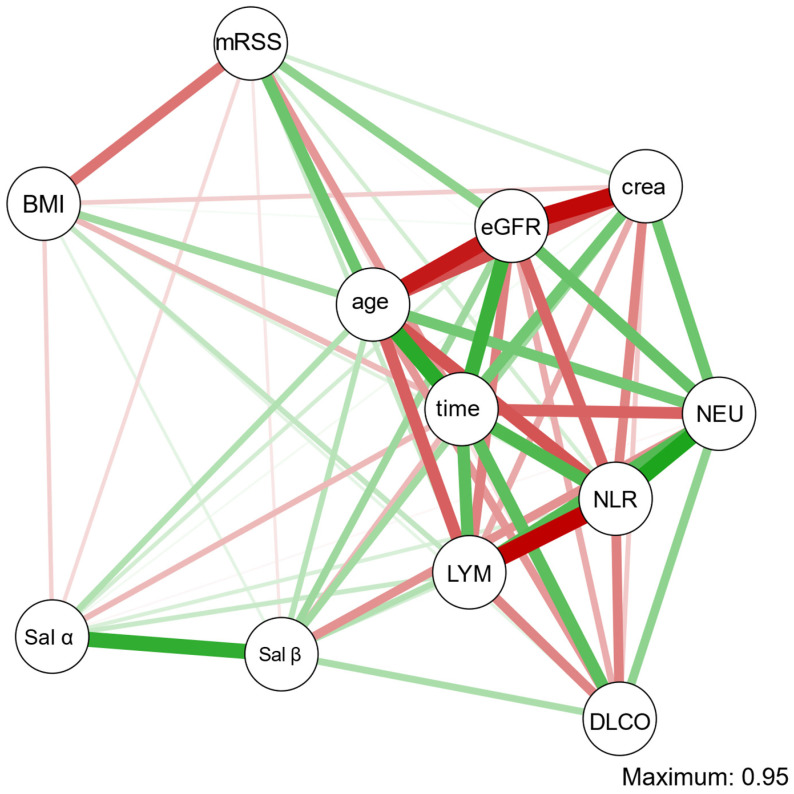
Gaussian graphical model for continuous variables evaluated in the experimental group. Legend: crea—serum creatinine concentration; DLCO—diffusion lung capacity for carbon monoxide; time—disease duration; eGFR—estimated glomerular filtration rate; LYM—lymphocyte percentage; mRSS—modified Rodnan scale score; NLR—neutrophil to lymphocyte ratio; NEU—neutrophils percentage; Sal α—serum salusin-α; Sal β—serum salusin-β. Green color—positive partial correlation; red color—negative partial correlation; thickness of the line reflects the strength of the correlation.

A moderately strong negative correlation was found between serum creatinine levels and the concentrations of salusin-α (Spearman’s ρ = −0.32, *p* = 0.035). Similar results were obtained for salusin-β (Spearman’s ρ = −0.37, *p* = 0.015). No statistically significant correlations or associations were detected between salusins concentrations and parameters reflecting disease involvement of skin or other organs in systemic sclerosis (Table 3, Figure 3).

### 3.4. Comorbidities

Diabetic patients had significantly decreased salusin-β levels (123.95 [113.7–134.2] vs. 174.5 [116.9–3288.0] pg/mL, U = 5.0; *p* = 0.037); however, the subgroup was represented by two patients only. No such observation was made for salusin-α (151.35 [128.2–174.5] vs. 187.0 [85.7–951.8] pg/mL, U = 19.0, *p* = 0.172). No correlations were observed between the presence of thyroid disease (either hypo- or hyperthyroidism) and salusin-α (174.7 [97.4–951.8] vs. 182.2 [85.7–823.0] pg/mL, U = 185.0, *p* = 0.659) or β concentrations (174.5 [121.8–1192.0] vs. 167.9 [113.7–3288.0] pg/mL, U = 185.0, *p* = 0.753). No statistically significant association was detected when the occurrence of other autoimmune disorders (such as lichen planus, vulvar lichen sclerosus, cicatricial alopecia, Sjögren syndrome, antiphospholipid syndrome, and primary sclerosing cholangitis) was compared with salusin-α (204.5 [152.6–823.0] vs. 180.8 [85.7–951.8] pg/mL, U = 162.0, *p* = 0.372) or salusin-β serum levels (192.2 [134.2–1840.0] vs. 167.9 [113.7–3288.0] pg/mL, U = 164.0, *p* = 0.400).

### 3.5. Treatment

**Figure 4:**
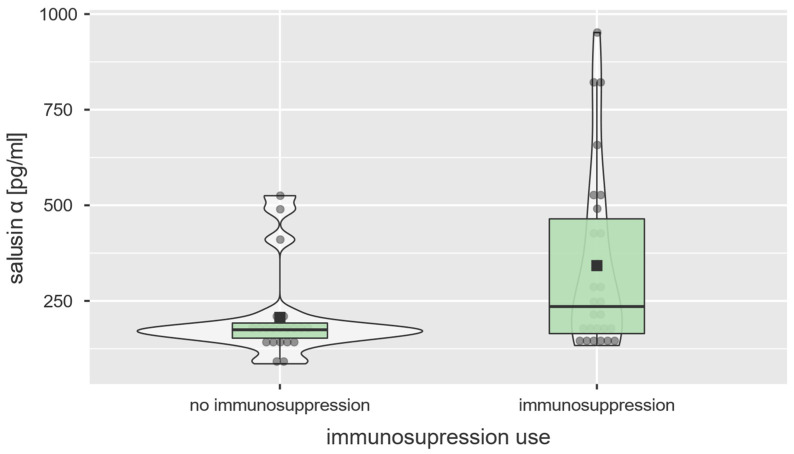
Association between serum salusin-α levels [pg/mL] and therapy used (U = 176.0; *p* = 0.026).

Selected patients were treated with single-drug immunosuppression (n = 27) together with oral medications of rheologic properties (pentoxifylline, sildenafil, or oral sulodexide in various combinations) on a daily basis or intravenous ones (alprostadil, epoprostenol, or sulodexide) while admitted to the hospital. Immunosuppressive medications used by the patients included mycophenolate mofetil (n = 16), methotrexate (n = 10), and cyclophosphamide (n = 1). Patients not receiving immunosuppression therapy, were prescribed with rheologic medications only. Patients undergoing immunosuppressive treatment had statistically significantly higher salusin-α concentrations than those on rheologic therapy only (235.3 [133.5–951.8] vs. 174.5 [85.7–525.1] pg/mL, U = 176.0; *p* = 0.026, Table 4, Figure 4). No similar associations were found between treatment modalities and salusin-β concentrations (281.2 [121.2–2399.0] vs. 156.6 [113.7–3288.0] pg/mL, U = 191.0; *p* = 0.081).

## 4. Discussion

In our study, we compared the concentrations of salusin-α and salsin-β between the groups of patients with systemic sclerosis and healthy volunteers. We found statistically significant elevation of salusin-α in individuals with systemic sclerosis (*p* = 0.004; Figure 1, Table 2). Such a difference was not detected in regard to salusin-β. Furthermore, we showed that both salusins were strongly correlated with each other (*p* < 0.001, R^2^ = 0.57), which is consistent with their common origin from one precursor peptide. As expected, their concentrations were lower and better linearly correlated in healthy volunteers (*p* < 0.001; R^2^ = 0.93) than in patients with systemic sclerosis (*p* < 0.001; R^2^ = 0.55), in whom it is hypothesized that these mediators may play a role in disease pathogenesis leading to their higher concentrations and noticeable balance disturbances.

A moderately strong negative association between serum creatinine and salusin-α (Spearman’s ρ = −0.32, *p* = 0.035) and β (Spearman’s ρ = −0.37, *p* = 0.015) was reported; however, a potential clinical association could not be identified. This finding may be related to renal function of the patients, as salusins are also produced in the kidneys. Nevertheless, none of the patients had a documented history of current acute kidney injury (AKI) or end-stage renal disease (ESRD).

We also noted that a statistically significant correlation was present between salusin-α levels and the therapy used. Patients undergoing immunosuppressive treatment had significantly higher salusin-α concentrations than those treated with vasodilators only (*p* = 0.026; Figure 4, Table 4). This phenomenon associated with pharmacologic treatment has not been observed in previous studies on salusins. Furthermore, no previous studies analyzing both salusin-α and salusin-β concentrations in systemic sclerosis were published to date.

As potential peptide biomarkers, salusins are being studied since 2003 [9]. Despite that, they are not commonly measured in patients beyond research as their role and usefulness in clinical practice has not yet been fully established. Decreased salusin-α and elevated salusin-β reflecting endothelial disfunction were primarily observed in patients with chronic kidney disease and cardiovascular diseases, such as coronary artery disease, atherosclerosis, and hypertension [10,23,37,38]. In a recent study, salusin-α was found to be negatively correlated with the cardiovascular risk score of the newly validated systematic coronary risk estimation 2 (SCORE2) algorithm [39,40]. Some reports indicate the role of salusins in diseases of dermatologic and/or rheumatologic origin.

### 4.1. Salusin Concentration in the Skin and Connective Tissue Diseases

The literature regarding the role of salusins in patients with dermatological and rheumatological disorders is inconsistent. In systemic sclerosis and systemic lupus erythematosus (SLE), Koca et al. [41] observed decreased salusin-α concentrations. Nevertheless, after adjusting for age, no correlation was identified between salusin-α concentrations and disease activity, intima media thickness, or TNFα and IL-6 levels. Erden et al. [42,43] also observed decreased salusin-α concentrations in psoriasis and Behçet disease. Importantly, salusin-α was even lower in patients with metabolic syndrome and, regarding psoriasis, also in individuals with higher psoriasis area severity index (PASI) score.

Elevated salusin-α concentrations were reported by Ozgen et al. [44] in a group of patients with rheumatoid arthritis and Behçet’s disease, which were also positively correlated with the intima media thickness (after adjusting for age, gender, BMI, and current treatment). Kobak et al. [45] also observed significantly elevated salusin-α concentrations in psoriatic arthritis, which positively correlated with CRP values and arthritis.

As previously mentioned, salusin functions and concentrations were well-investigated in mice models and in vitro studies. Nonetheless, the reports in skin and connective tissue diseases involving humans stay inconclusive due to the complexity of occurring processes and misidentified confounders such as pharmacotherapy and comorbidities [46].

### 4.2. The Use of Corticosteroid Therapy

An important factor not considered in previously discussed reports on salusins was the frequent use of long-term systemic corticosteroid therapy, which was an exclusion criterion in our study. Long-term corticosteroid intake is considered to be a strong confounding factor due to possible influence on various physiological and inflammatory processes; therefore, the association between the biomarker concentration and the treatment used may be potentially affected. In our study, patients receiving oral treatment with corticosteroids as well as other factors alleviating immune responses (such as oral non-steroidal anti-inflammatory drugs or pregnancy) were not enrolled due to potential interference with inflammatory parameters (e.g., CRP and lymphocyte count) and, consequently, biomarker concentrations.

Erden et al. [43] showed decreased salusin-α levels in Behçet disease; however, 52% of patients were undergoing treatment with corticosteroids together with non-corticosteroid immunosuppressants such as azathioprine, colchicine, and cyclosporine. Koca et al. [41], who observed no statistically significant decrease of salusin-α in systemic sclerosis and systemic lupus erythematosus, indicated that 95% of the patients with systemic lupus erythematosus and 36% of patients with systemic sclerosis were receiving systemic corticosteroids. Kobak et al. [45] reported statistically significant elevated salusin-α levels in psoriatic arthritis; however, 15% of patients received low-dose corticosteroids and others were treated with methotrexate, biologic agents, or did not receive any therapy.

In Behçet disease, Ozgen et al. [44] performed a separate analysis regarding the treatment type and observed some statistically significant correlations. In that study, salusin-α was significantly elevated in patients receiving systemic corticosteroids (corresponding to 30.6% of the experimental group), while salusin-α was decreased in those treated with azathioprine (accounting for 22.2% of the treatment group) [44].

### 4.3. Non-Corticosteroid Immunosuppressive Medications

In our study, we observed a statistically significant correlation between immunosuppression and antiatherogenic salusin-α–patients receiving immunosuppressive medications with higher salusin-α concentrations. The anti-inflammatory and atheroprotective properties of selected non-corticosteroid immunosuppressive agents were reported in the literature [47].

The immunosuppressive medication with the widely proven impact on endothelial disfunction is methotrexate [48,49]. Methotrexate was shown to attenuate the secretion of proinflammatory mediators and monocyte adhesion in mice cells via an adenosine-related pathway [50,51]. In result, the processes of vascular inflammation involving T-cell mediated cytotoxicity, neutrophil oxidative burst, and NF-κB pathways associated with reactive oxygen species production are inhibited [48]. In a 12-week open-label study on patients with rheumatoid arthritis, methotrexate was also reported to enhance adenosine-induced vasodilation (determined by forearm blood flow measurement and adenosine deaminase activity in erythrocytes and lymphocytes) [52]. In another study involving patients treated with methotrexate for rheumatoid arthritis, a clinical difference was shown in carotid intima media thickness [53,54], while other studies did not shown any significant changes in this parameter or altered aortic distensibility [55,56].

Furthermore, complex anti-atherosclerotic and anti-inflammatory properties of mycophenolate mofetil metabolite were described [57], i.e., via limiting the in vitro adhesion of mononuclear leukocytes to endothelial cells [58] and inhibiting fibroblast nitric oxide synthase [59,60]. Mycophenolate mofetil also inhibits TNF-α-induced NF-kB activation, which results in attenuated reactive oxygen species production [61]. Nevertheless, one study on patients with systemic lupus erythematosus has not shown inhibition of atherogenesis and indicated progression of intima media thickness over a 2-year period despite mycophenolate mofetil therapy in various dosages [62].

Brief reports are available regarding the atheroprotective properties of cyclophosphamide. In a low dose, cyclophosphamide was shown to inhibit atherosclerosis in murine model by a reduction of Th1 response and monocyte/macrophage migration to the plaque [63].

It can be hypothesized that vasodilative and anti-inflammatory processes inducted by non-corticosteroid immunosuppressive agents could possibly induce salusin-α production and, in result, enhance other processes against endothelial disfunction in systemic sclerosis. Nonetheless, the association between immunosuppression and endothelial function is not confirmed due to existing research gaps and the complexity of autoimmune processes in humans in comparison to in vitro studies [46].

## 5. Study Limitations

The main limitation of this study is a small sample size, which confines the ability to identify all true correlations and results in the small number of some secondary endpoints (e.g., only one patient had pulmonary hypertension). No significant correlations were detected between salusin-β levels and other preselected parameters, which may also result from small sample size or previously reported fluctuating concentrations due to the adherence of salusin-β to plastic laboratory tubes [64]. This study had a retrospective exploratory character, which results in the presence of missing data and does not allow for drawing causative inference.

## 6. Conclusions

Salusins are circulating peptide biomarkers of endothelial disfunction. High serum salusin-α is a prospective marker of the preserved function of endothelial cells, while high serum salusin-β indicates the occurrence of vascular remodeling. To our knowledge, this is the first study investigating concentrations of both salusin-α and salusin-β in systemic sclerosis.

In this study, elevated salusin-α concentrations were observed in patients with systemic sclerosis compared with healthy individuals (*p* = 0.004). Furthermore, systemic sclerosis patients receiving immunosuppressive treatment had significantly higher salusin-α concentrations than those treated with rheologic medications only (*p* = 0.026). Both salusins levels were statistically significantly correlated with serum creatinine concentrations.

Due to the limited evidence regarding salusins in the skin and connective tissue diseases, additional research is needed to fully understand the hypothesized positive impact of salusin-α in pharmacologically treated patients with systemic sclerosis.

## Figures and Tables

**Table 1:** Demographic, clinical, and laboratory results of patients in the experimental group.

Variable	Patients with Systemic Sclerosis (N = 48)
women, n, %	44 (92%)
age, years	56.4 (11.4)
BMI, kg/m^2^	24.9 (4.1)
duration of disease since the first diagnosis, years	6.5 [1–38]
serum creatinine, mg/dl	0.73 [0.48–1.06]
CKD-EPI eGFR, mL/min/1.73 m^2^	92.78 (16.88)
mRSS	6 [2–22]
DLCO, n, %	71.1 [36.0–135.6]
neutrophiles, n, %	63.6 (8.7)
lymphocytes, n, %	25.2 (7.6)
NLR	2.63 [0.94–6.69]
coronary artery disease, n, %	7 (15%) *
hyper- or hypothyroidism, n, %	11 (23%)
arterial hypertension, n, %	17 (35%)
gastroesophageal reflux disease, n, %	11 (23%) *
another autoimmune disease, n, %	11 (23%) *
dyslipidemia, n, %	13 (30%) ᶧ
Scl-70 antibodies, n, %	23 (49%) *
ACA antibodies, n, %	19 (40%) *
immunosuppressive treatment, n, %	27 (57%) *

Legend: BMI—body mass index; CKD-EPI eGFR—estimated glomerular filtration rate calculated with the Chronic Kidney Disease Epidemiology Collaboration (2021 update) equation; DLCO—diffusing capacity of the lung for carbon monoxide; NLR—neutrophil to lymphocyte ratio; mRSS—modified Rodnan scale score. Results are reported as median [range] or mean (standard deviation), as appropriate. *—missing data in one patient. ᶧ—missing data in five patients.

**Table 2:** Salusin concentrations in the studied groups.

Salusin	Patients with Systemic Sclerosis (N = 48)	Healthy Volunteers (N = 25)	Test Statistic and *p* Value
salusin-α, pg/mL, median [range]	181.85 [85.66–951.80]	120.70 [24.03–362.40]	U = 350.5, *p* = 0.004
salusin-β, pg/mL, median [range]	173.70 [113.70–3288.00]	186.30 [120.30–773.50]	U = 550.0, *p* = 0.662

**Table 3:** Correlations or associations between parameters reflecting skin or internal organ involvement in systemic sclerosis and salusins concentrations.

Organ/System	Variable	For Salusin-α		For Salusin-β	
		Test Statistic/Correlation Coefficient	*p* Value	Test Statistic/Correlation Coefficient	*p* Value
**skin**	mRSS	ρ = −0.05	0.772	ρ = −0.12	0.474
	fingertip ulcerations	U = 71.0	0.101	U = 65.0	0.150
**cardiovascular**	Raynaud’s phenomenon	U = 228.5	0.227	U = 184.5	0.053
	arterial hypertension	U = 244.5	0.690	U = 242.0	0.782
	coronary artery disease	U = 112.5	0.420	U = 115.0	0.521
**pulmonary**	lung fibrosis in HRCT	U = 92.0	0.724	U = 99.0	0.986
	DLCO	ρ = 0.12	0.561	ρ = 0.22	0.305
**gastrointestinal**	esophageal dysmotility in X-ray with contrast	U = 65.5	0.461	U = 54.5	0.267
	gastroesophageal reflux	U = 194.0	0.930	U = 183.5	0.725
**renal**	serum creatinine	ρ = −0.32	0.035	ρ = −0.37	0.015

Legend: DLCO—diffusing capacity of the lung for carbon monoxide; HRCT—high-resolution computed tomography; mRSS—modified Rodnan scale score; ρ—Spearman’s rank correlation coefficient.

**Table 4:** Salusin-α concentrations in the experimental group—patients treated with either vasodilators only or with vasodilators in combination with immunosuppressive agents.

No Immunosuppression (N = 17), pg/mL, Median [Range]	With Immunosuppression (N = 27), pg/mL, Median [Range]	Test Statistic and *p* Value
174.50 [85.66–525.10]	235.30 [133.50–951.80]	U = 176.0, *p* = 0.026

## Data Availability

Not Applicable.
